# 3D bioprinting of a corneal stroma equivalent

**DOI:** 10.1016/j.exer.2018.05.010

**Published:** 2018-08

**Authors:** Abigail Isaacson, Stephen Swioklo, Che J. Connon

**Affiliations:** Institute of Genetic Medicine, Newcastle University, Newcastle Upon Tyne, UK

**Keywords:** 3D bioprinting, Tissue engineering, Cornea, Keratocytes, Collagen, Bio-ink

## Abstract

Corneal transplantation constitutes one of the leading treatments for severe cases of loss of corneal function. Due to its limitations, a concerted effort has been made by tissue engineers to produce functional, synthetic corneal prostheses as an alternative recourse. However, successful translation of these therapies into the clinic has not yet been accomplished. 3D bioprinting is an emerging technology that can be harnessed for the fabrication of biological tissue for clinical applications. We applied this to the area of corneal tissue engineering in order to fabricate corneal structures that resembled the structure of the native human corneal stroma using an existing 3D digital human corneal model and a suitable support structure. These were 3D bioprinted from an in-house collagen-based bio-ink containing encapsulated corneal keratocytes. Keratocytes exhibited high cell viability both at day 1 post-printing (>90%) and at day 7 (83%). We established 3D bio-printing to be a feasible method by which artificial corneal structures can be engineered.

## Graphical abstract

**Figure:**
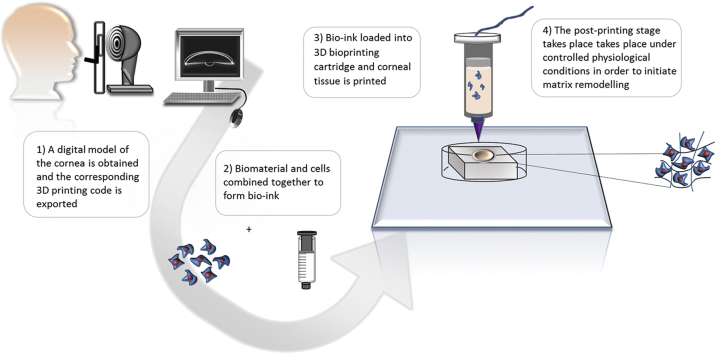

## 1 Introduction

The World Health Organisation estimates that 10 million people worldwide require surgery to prevent corneal blindness as a result of trachoma, with a further 4.9 million suffering from total blindness due to corneal scarring (Whitcher et al., 2001). Even with adequate numbers of prospective cornea donors, a considerable discrepancy exists between the supply and demand of transplantable corneas (Golchet et al., 2000). The unmet clinical need for cornea donors has led to increasing effort in the development of artificial corneal substitutes, which must meet specific criteria if they are to functionally mimic the native cornea.

The cornea serves as the protective, outermost layer of the eye and is responsible for the transmission and refraction of incident light beams that are in turn focused onto the retina by the lens (Eghrari et al., 2015; Meek and Knupp, 2015; Griffiths et al., 2016). Its near-perfect spherical anterior surface, together with the index of refraction change at the air/tear film interface, account for approximately 80% of the total refractive power of the human eye (Ruberti and Zieske, 2008). The ability to recapitulate the rotational symmetric curvature necessary for optical refractive power is therefore fundamental to the design framework that exists for engineering functional corneal substitutes (Muller et al., 2001; Ruberti and Zieske, 2008).

It is the distinct arrangement of collagen lamellae in the corneal stroma that is responsible for maintaining the strength and shape of the cornea (Farrell and McCally, 2000). The corneal stroma comprises somewhere between 200 and 250 lamellae that are assembled heterogeneously throughout its depth, with the lamellae in the mid to posterior stroma lying in parallel while those in the anterior stroma are interwoven with one other (Hogan et al., 1971). The complexity of corneal microstructure presents an ongoing challenge when using traditional tissue engineering methods that focus on assembling corneal extracellular matrix (ECM) in vitro. As such, the replication of human corneal geometry remains to be fully realised within the context of tissue engineering.

The ability to construct biosynthetic corneal models would be useful for a number of applications, and this has been achieved in recent years where, for example, corneal models have been required for the characterisation of corneal cellular regeneration (Li et al., 2003) as well as for modelling corneal fibrosis (Karamichos et al., 2014). In these instances, corneal models were assembled using different techniques; the study by Li et al. made use of plastic contact lens molds into which hybrid collagen hydrogels were injected and crosslinked, while Karamichos et al. plated human corneal fibroblasts (HCFs) onto six-well plates bearing porous polycarbonate membrane inserts that were left in culture over a period of weeks. The former method enabled the fabrication of a curved corneal surface onto which corneal epithelial cells could be seeded, cultured and eventually implanted, while the latter relied upon cell proliferation and ECM secretion to render an in vitro 3D model that could be used to study fibrosis reversal in the cornea.

3D bioprinting is a technique that has garnered notable interest for tissue engineering applications for its ability to direct the hierarchical assembly of 3-dimensional biological structures for tissue construction (Mironov et al., 2006). Since its advent, 3D bioprinting has made possible the layer-by-layer deposition of biological materials in a prescribed pattern corresponding with the anatomy of an organotypic model (Zhang and Zhang, 2015). This model is usually acquired from clinical images such as CT and MRI scans and is used as a means by which to generate the fundamental printing paths on which 3D bioprinting depends; these are expressed in the form of a unique G-code that can be computed automatically by 3D printing software at resolutions specified by the chosen print parameters (Murphy and Atala, 2014). The ability to replicate features such as concavity, undercuts and convoluted patterns is therefore a function of the complexity of two-dimensional figures, such as points, lines and circles (Chia and Wu, 2015). The final, post-printing stage involves the cell-mediated remodelling of the printed biological construct in the presence of appropriate physiological cues to ensure that it develops suitable biomechanical, structural and functional properties (Jakab et al., 2010; Mironov et al., 2011).

In this study, we examined the feasibility of generating complex 3D bioprinted corneal stroma equivalents using pneumatic 3D extrusion bioprinting. Printed constructs were anatomically analogous to a human corneal model derived from the topographic data of an adult human cornea, acquired *in situ* post-refractive surgery. Several low viscosity bio-ink combinations were tested for their printability prior to cell incorporation. Printing accuracy was evaluated by quantifying central and peripheral thickness of the corneal construct and the viability of encapsulated corneal keratocytes was evaluated on days 1 and 7 post-printing. Overall, our study provides a basis for further research into the use of 3D bioprinting for the generation of artificial, biological corneal structures for regenerative medicine applications.

## 2 Materials and methods

### 2.1 Digital model and support structure generation

A patient-specific digital corneal model constructed using a rotating Scheimpflug camera with a Placido disk and discretised by the Finite Element Method (FEM) was used (Simonini and Pandolfi, 2015). The vertical and horizontal diameters of the model measured 12.377 mm × 12.385 mm, respectively, while its thickness measured approximately 500 μm at the centre and 823 μm towards the periphery. The corneal model was used as a template with which to build a digital support structure on AutoCAD 2017 (version 20.1) in order to facilitate the 3D bioprinting process. This was made possible by sealing the rim of the model cornea with a planar circle (r = 6.5 mm) such that it then resembled a dome; the modified model was then subtracted from the centre of one of the square faces of a digital cuboid (23.7 mm × 23.7 mm x 6 mm) that was designed to sit neatly inside a 35 mm Petri dish. The resulting support structure was exported as an STL file and 3D-printed at a resolution of 100 μm with white Acrylonitrile Butadiene Styrene (ABS) using a CEL Robox 3D printer.

### 2.2 G-code export and printing setup

The 3D printing software Slic3r (1.2.9) was configured with an INKREDIBLE bioprinter (Cellink AB, Gothenburg, Sweden). A stereolithography (STL) file of the corneal model was imported onto Slic3r, from which versions of G-code were subsequently exported. Printing speed was set at 6 mms^−1^ and 30G high precision blunt needles (CELLINK, AB) were used in all experiments.

### 2.3 Bio-ink preparation

Sodium alginate (Acros Organics brand, ThermoFisher Scientific, U.K.) and methacrylated type I collagen (PhotoCol^®^, Advanced Biomatrix, USA) were used to prepare all bio-inks. Methacrylated collagen was first dissolved in acetic acid and subsequently neutralized with sodium hydroxide at 4 °C, following the supplied preparation protocol. Six bio-inks were formulated in total, two of which comprised 3% (w/v) sodium alginate and 8 mg/ml methacrylated collagen only. The final four bio-inks, termed Coll-1 to Coll-4, had various combinations of methacrylated collagen mixed with 2% (w/v) sodium alginate to the following ratios: (i) Coll-1: one part 8 mg/ml collagen to two parts alginate; (ii) Coll-2: one part 8 mg/ml collagen to three parts alginate, (iii) Coll-3: one part 6 mg/ml collagen to two parts alginate; and (iv) Coll-4: one part 6 mg/ml collagen to three parts alginate.

### 2.4 Cell culture

Corneal keratocytes are the most abundant cell type in the corneal stroma, itself comprising 80% of corneal thickness, and were therefore deemed a suitable cell type for bio-ink formulation. Human corneal stromal cells were isolated, as previously described (Gouveia and Connon, 2013), from cadaverous human corneal tissue (male/female, age 60–80 years and with no prior history of corneal diseases or ocular trauma, research consent given) obtained from NHS Blood and Transplant (NHSBT) through a service level agreement with Newcastle-upon-Tyne Hospitals NHS Foundation Trust, U.K.. Briefly, the epithelia-depleted corneal tissues were finely chopped using a scalpel, transferred to DMEM/F12 medium (ThermoFisher Scientific) supplemented with 5% fetal bovine serum (FBS; BioSera, Labtech International, U.K.), 2 g/L (450 units/mL) collagenase type-1 (ThermoFisher Scientific) and incubated at 37 °C under continuous rotation for 5 h, followed by incubation with 0.25% trypsin-EDTA (ThermoFisher Scientific) for 10 min. The isolated corneal stromal cells were plated onto tissue culture flasks (Greiner Bio-One, U.K.) and maintained using DMEM/F12 medium supplemented with 5% FBS and 1% penicillin/streptomycin (ThermoFisher Scientific). Media were changed every 2–3 days, and cultures were maintained until reaching 70–80% confluence. At this point fibroblasts underwent serum starvation for a period of 3 days to promote their differentiation into keratocytes. During this time, cells were cultured in serum-free medium comprised of DMEM/F12 with 1 × 10^−3^ M ascorbic acid (Sigma-Aldrich, U.K), 1 × ITS (Sigma-Aldrich), and 1% penicillin/streptomycin. Cells were used at passage 3.

### 2.5 3D bioprinting and optimisation

A pneumatic, dual extruder 3D bioprinter was used to print corneal stromal equivalents. This was calibrated so that bioprinting began from the centre of the support and then outwards and upwards towards the rim. Following calibration, the hollowed-out section of the bespoke 3D printed plastic support was filled with gelatine slurry in order to facilitate the printing of low viscosity collagen and alginate bio-inks while maintaining printability; the gelatine slurry was prepared using the Freeform Reversible Embedding of Suspended Hydrogels (FRESH) starter kit and protocol provided by Allevi (USA). The support was then returned to the printing plate for the duration of the printing process. Corneal structures were extruded at air pressures of 180 KPa, 15 KPa, 40 KPa, 20 KPa, 15 KPa and 10 KPa for bio-inks comprising 3% alginate alone, 8 mg/ml methacrylated collagen alone, Coll-1, Coll-2, Coll-3 and Coll-4, respectively. A phosphate buffered saline (ThermoFisher Scientific) (PBS)-based slurry and a calcium chloride (Sigma-Aldrich) (CaCl_2_)-based slurry were prepared for use with collagen and alginate bio-inks respectively, with the latter additionally used for the printing of composite bio-inks. Corneal structures printed using alginate-based bio-inks were crosslinked with 100 μl of 1% (w/v) CaCl_2_ and were immediately incubated at 37 °C for 8 min, while structures printed from collagen alone were incubated for 30 min at 37 °C. The gelatine slurry was aspirated after incubation leaving behind printed and stabilised constructs that were then detached from the support and stored in PBS thereafter. Corneal structures printed with composite bio-inks were crosslinked in 100 μl CaCl_2_ and incubated at 37 °C for 8 min; the CaCl_2_ was then aspirated and replaced with an equal volume of PBS and incubated for a further 20 min at 37 °C. Serum-free medium was used in place of PBS once cells were incorporated into the bio-ink. After printability had been optimized, corneal keratocytes were incorporated into Coll-1 at a concentration of 2 million cells/ml. These were printed using a nozzle diameter of 200 μm and were maintained in serum-free medium for 7 days subsequent to printing.

### 2.6 OCT sectioning

Prior to cell incorporation, composite corneal structures were printed with Coll-1 at nozzle diameters of 200 μm and 300 μm, embedded in optical cutting temperature gel and frozen for 1 h. Cross-sectional slices taken through the centre were imaged using a Leica DM IL LED microscope (Leica, UK) and the thickness at both the centre and the periphery were measured using ImageJ (1.48v) software (Schneider et al., 2012).

### 2.7 Cell viability evaluation

Viable cell number and percentage viability of corneal keratocytes were assessed using a Countess II FL automated cell counter (Invitrogen brand, ThermoFisher Scientific). Corneal structures were stained with 1 μM Calcein-AM (eBioscience brand, ThermoFisher Scientific) and 2 μM Ethidium Homodimer 1 (Sigma-Aldrich) at 37 °C for 15 min. Fluorescent images were captured on days 1 and 7 post-printing using a Leica DM IL LED microscope (Leica, UK) from which cell viability was calculated using ImageJ (1.48v) software (Schneider et al., 2012). Briefly, RGB images of separate channels were first converted to binary images to enable the counting of cells by analysing particle number. The range of particles to be counted was chosen based on area measurements of the smallest and largest observed particles on each binary image. Percentage viability was calculated as the number of live cells, divided by the total number of cells (live and dead), multiplied by 100.

## 3 Results

### 3.1 Support structure generation

**Fig. 1:**
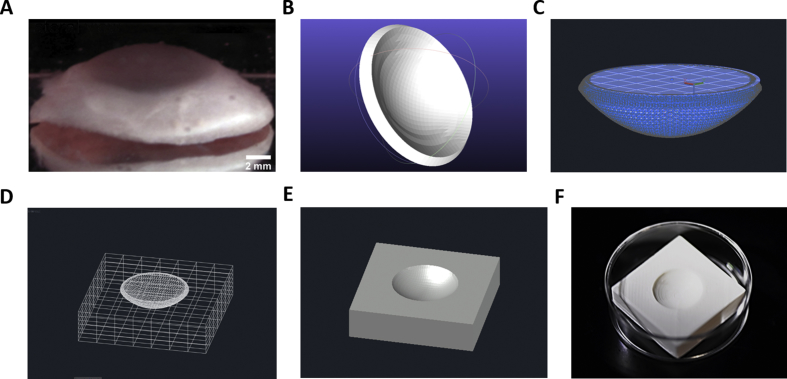
Stages of support structure generation. (A) Human cornea showing size and natural curvature across surface. (B) Original corneal model derived by (Simonini and Pandolfi, 2015) via the Finite Element Method (FEM). Corneal model is first converted to a solid to enable the execution of Boolean operations required for the generation of a support structure. Cornea is then ‘sealed’ (C) with a planar circle in order to subtract its volume from the support structure. (D) Wireframe view of cornea situated at the centre of cuboid prior to subtraction. (E) Digital support structure after subtraction. (F) The 3D printed plastic support structure.

The geometry of a digital corneal model was required for the generation of 3D printed corneal structures. This was obtained previously by the acquisition of cornea elevation maps and a point cloud defining the anterior corneal surface, followed by the evaluation of missing points by interpolation and the subsequent reconstruction and discretization of the cornea into finite elements (Simonini and Pandolfi, 2015). A support structure was additionally required in order to preserve the shape of printed corneal constructs during and after bioprinting. This was obtained by constructing a digital image that exhibited a geometry complementary to that of the anterior corneal surface. The SURFSCULPT command on AutoCAD was used to transform the original cornea model together with a planar circle into a solid dome structure (Fig. 1). The modified cornea was then positioned just above the surface of a cuboid at its widest diameter to enable printing; this was followed by the subtraction of its entire volume, generating a structure that could support the printing of corneal constructs (Fig. 1). The support structure was designed to closely fit inside a 35 mm petri dish to facilitate the transfer of the constructs for immediate incubation (Fig. 1). Importantly, there was no interference between the needle and the support structure during printing.

### 3.2 3D bioprinting optimisation and evaluation of cornea-like structures

**Fig. 2:**
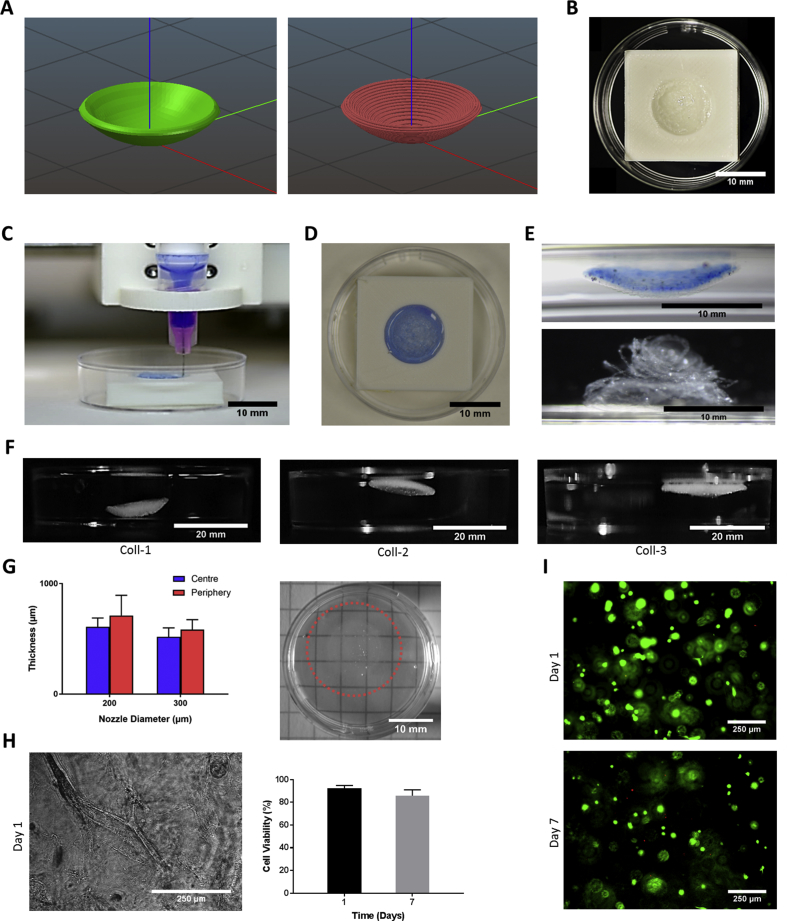
Using support structure to facilitate the printing of a corneal structure with 3% alginate (nozzle diameter = 200 μm) and optimisation of bio-inks for corneal 3D bioprinting. (A) Digital cornea is imported to the computer driving the 3D printer software slic3r and a preview of the concentric directionality of print is displayed. (B) The support structure is coated with FRESH to facilitate the 3D bioprinting of corneal structures. (C) View of the 3D bioprinting process. Corneal structures were printed with 3% alginate bio-ink stained with trypan blue to increase visibility. (D) Image of 3D bioprinted corneal structure captured prior to incubation. (E) FRESH is aspirated after 8 min of incubation and corneal structure is carefully removed from support, but begins to unravel 1 day post-printing once keratocytes were combined with the alginate bio-ink. (F) Images of corneal structures 3D bioprinted from composite bio-inks. (G) Relationship between nozzle diameter and printed thickness of corneal structures (left) and depiction of transparency of corneal structure 3D bioprinted from Coll-1 bio-ink (H) Brightfield image of 3D bioprinted corneal structure containing cells at day 1 (left) and cell viability measurements over 7 days (right). (I) Representative live/dead stain images using fluorescence microscopy at days 1 and 7 after 3D bioprinting in Coll-1.

In order to print corneal structures, the original model cornea was imported onto Slic3r so that versions of corresponding G-code could be exported. Print directionality was set to concentric as this resulted in the least disruption of deposited bio-ink. Similarly, a straight profile needle was used in order to prevent possible disruption of the gelatine slurry and over-dispensation of bio-ink (Fig. 2). Structures printed from 3% (w/v) sodium alginate bio-ink were sufficiently stiff such that corneal curvature was maintained after detachment from the support. Corneal constructs printed in the absence of the gelatine slurry prescribed by the FRESH method either did not maintain their printed shape, or, as was the case when a more viscous bio-ink was used, clumped together as a result of the concentric movements of the needle. Importantly, no needle blockage occurred and the gelatine slurry was displaced to accommodate the bio-ink while simultaneously supporting the formation of the corneal structure layer by layer. Following crosslinking by addition of CaCl_2_, the resulting corneal structures detached from the plastic support without difficulty and retained their shape after transferal into PBS. However, when corneal keratocytes cells were incorporated into the alginate bio-ink the resulting printed corneal structures began to unravel with minimal mechanical stimulation (Fig. 2). To rectify this we first tried a collagen bio-ink in place of alginate, but found that concentrations suited to our extrusion system were not viscous enough i.e. unable to hold their shape once extruded or the printed threads would diffuse through gelatine. We therefore set about formulating a set of novel composite bio-inks comprising both collagen and alginate in order to combine their respective material and mechanical properties and, in doing so, optimise the print fidelity and mechanical integrity of corneal structures.

### 3.3 Formulation and evaluation of composite bio-inks to print corneal structures

Four composite bio-inks were formulated that incorporated combinations of type I collagen and sodium alginate in varying proportions. Structures printed with Coll-4 presented little structural integrity and fragmented easily whereas those printed with Coll-3 remained intact but were unable to maintain an appropriate degree of curvature (Fig. 2). Thus, the concentration of incorporated collagen was then raised to improve printability and strength. Structures printed with Coll-2 displayed more curvature than those printed with Coll-3, while the best preservation of corneal shape was obtained with the Coll-1 bio-ink (Fig. 2). The enhanced stability of the corneal structures printed with Coll-1 was attributed to the combined tensile strength of collagen and alginate hydrogel stiffness post-crosslinking. Composite Coll-1 corneal structures also demonstrated an improved degree of transparency (compared to the collagen only bio-inks), which was aided by the transparency of incorporated alginate (Fig. 2). Owing to its printability, Coll-1 bio-ink was subsequently used to assess the effect of nozzle diameter on print accuracy.

Due to the shortened printing path that is generated when nozzle diameter is increased, central and peripheral corneal thickness was significantly reduced when a nozzle diameter of 300 μm was applied relative to the 200 μm nozzle due to the decrease in bio-ink deposition (Fig. 2). The central and peripheral thickness of the 3D bioprinted constructs were recorded as 609.4 μm and 711.2 μm for the 200 μm nozzle, and 518.5 μm and 584.7 μm for the 300 μm nozzle, respectively. Further experiments were conducted with a 200 μm nozzle setting as these structures exhibited greater stability due to the additional points of integration between adjacent layers of filament. Subsequent to cell incorporation, high initial viability of corneal keratocytes was observed on day 1 post-printing (92%) with discernible cell spreading and no cell aggregates (Fig. 2). Cell viability remained high after 7 days at 83%.

## 4 Discussion

3D bioprinting is an additive manufacturing technology whereby cells are combined with a suitable biomaterial and deposited within micrometer precision, layer-by-layer, in order to generate tissue constructs for a variety of applications, including but not limited to tissue engineering (Mandrycky et al., 2016). Much of its appeal lies in its high-throughput ability, enabling the rapid fabrication of tissue scaffolds with high definition. Pneumatic extrusion bioprinting in particular enjoys a simple driving mechanism whereby its operating force is determined solely by the air-pressure capabilities of the system, where it has been shown to accommodate material viscosities as low as 30 mPa/s (Murphy and Atala, 2014).

Corneal 3D bioprinting necessitates the use of low viscosity biomaterials for the physical extrusion of bio-ink at high resolutions. The incorporation of a method that facilitates the 3D bioprinting of low viscosity bio-inks by preserving their intricate form during bio-ink dispensation was therefore fundamental. In this study, we demonstrated that keratocyte-laden corneal stromal equivalents can be 3D bioprinted using specially developed low viscosity bio-inks at high resolutions using the FRESH method.

We established that the presence of both a gelatine slurry and a support structure was ideal for the fabrication of anatomically robust corneal structures. The use of a gelatine slurry for 3D bioprinting applications was first described by Hinton et al. who noted that the gelatine microparticles behave as a rigid body at low shear stresses yet flow as a viscous fluid at high shear stresses, thus accommodating the continuous extrusion of bio-ink while retaining the shape of previously deposited bio-ink (Hinton et al., 2015). In this study, we observed the gelatine slurry gradually migrate towards the centre of the support structure to make way for the construct being printed. We also noted that, in the absence of a suitable support structure, corneal structures did not retain their curvature after aspiration of the slurry and that this likely occurred due to the rapid dissolution of the small volumes required to hold the printed constructs in place during printing. A bespoke support structure with the inverse shape to the cornea model was therefore constructed and used in conjunction with and after bioprinting. Consequently, the gelatine solution could be aspirated and replaced with growth medium and/or cross-linked while the constructs were still being supported.

Collagen constitutes a major component of corneal ECM (Ruberti and Zieske, 2008; Cen et al., 2008) and therefore presents a natural choice for generating bioengineered corneal structures. Low concentrations of collagen do not however possess the necessary stiffness to enable the fabrication of robust corneal structures via an extruding 3D bioprinter, where precise control over microarchitecture presents a major challenge. Hydrogels have been extensively used for tissue engineering applications for their structural resemblance to the ECM, their low toxicity and tuneable biophysical properties (Lee and Mooney, 2012; Drury et al., 2004; Klöck et al., 1997), including use in the cornea (Wright et al., 2012; Rafat et al., 2008; Guo et al., 2013). We undertook to compose a range of composite bio-inks comprising both collagen and alginate in an effort to combine the tensile strength of collagen with the biomechanical properties of alginate for the formation of printable corneal structures. Constructs printed with bio-ink Coll-1 in particular exhibited enhanced mechanical stability, which increased with the proportion of incorporated alginate.

The peripheral and central measurements acquired from tissue sectioning revealed the effect nozzle diameter had on print accuracy. Reduction in nozzle diameter resulted in a lengthening of the 3D printing path, giving rise to additional points of fusion between deposited filaments and an increase in mechanical stability. With all other print parameters remaining constant, a greater volume of bio-ink is invariably dispensed. Corneal constructs printed with a nozzle diameter of 200 μm appeared more robust, but better print fidelity was observed from those printed with a nozzle diameter of 300 μm. Thus, careful adjustment of print parameters such as printing speed, needle diameter and bio-ink viscosity can ensure both mechanical stability and print accuracy are achieved.

The all-encompassing challenge in 3D bioprinting concerns the ability to print intricate structures in which cells are able to retain viability without printability being compromised. Interpenetrating networks of alginate and collagen have previously been shown to provide a favourable environment suited to cell growth, where cells have manifested varying morphologies depending on material stiffness (Branco da Cunha et al., 2014). In this study, high initial viability of encapsulated corneal keratocytes in composite collagen and alginate bio-inks and noticeable spreading were observed. One of the limitations of extrusion 3D bioprinting is the generation of shear stress-induced cell deformation at the needle wall and which is diminished by the use of low viscosity bio-inks to which low air pressures can be applied (Ozbolat and Yu, 2013). The prevention of dehydration due to the presence of the gelatine slurry during printing and the thinness of the printed tissue also likely contributed to initial cell viability. The conservation of high cell viability of encapsulated cells 7 days after bioprinting points to the potential of composite collagen and alginate bio-inks for 3D bioprinting applications.

The future success of corneal 3D bioprinting will ultimately depend on the ability of encapsulated cells to mediate ECM remodelling in order to establish tissue functionality. A distinctive feature of corneal fibroblasts presents itself when they are seeded at the base of a curved surface where they have recently been shown to migrate in lattice formation and align collagen in a way that closely resembles its arrangement in the cornea (Gouveia et al., 2017). A significant advantage proffered by the present work is therefore the ability to reproduce curved corneal geometry which is now known to directly influence cell migration and collagen alignment. Thus, cells seeded at the base of a scaffold bearing a close resemblance to corneal anatomy would potentially be capable of remodelling the ECM in a way that is presently unachievable with non-curved geometries.

Successful translation of this current proof-of-concept study will first require further analysis of stromal cell phenotype, the biocompatibility of the construct following transplantation, its capacity to support epithelial cell growth and, critically, its ability to impart a functional corneal replacement. A significant benefit of this approach to corneal tissue engineering is that both structural and biochemical components of the equivalent can be rationally designed, allowing, in theory, the printing of anatomical features such as the limbal zone and Bowman's layer replete with appropriate soluble and insoluble factors.

## 5 Conclusion

In conclusion, our study provides a proof-of-concept for the use of 3D bioprinting as a rapid and effective method by which to fabricate human corneal substitutes from low viscosity bio-inks. Successful realisation of this method presently relies upon a sustained effort towards facilitating long-term matrix remodelling in order to validate clinical suitability. In all, these findings demonstrate great promise for the application of 3D bioprinting for corneal tissue engineering applications.

## Disclosure

The authors report no conflicts of interest.

## References

[bib1] FRESH Bioprinting Method. Allevi. https://biobots.io/biowiki/fresh-method/(accessed 26 December 2017).

[bib2] Branco da Cunha C., Klumpers D.D., Li W.A., Koshy S.T., Weaver J.C., Chaudhuri O., Granja P.L., Mooney D.J. (2014). Influence of the stiffness of three-dimensional alginate/collagen-I interpenetrating networks on fibroblast biology. Biomaterials.

[bib3] Cen L., Liu W., Cui L., Zhang W., Cao Y. (2008). Collagen tissue engineering: development of novel biomaterials and applications. Pediatr. Res..

[bib4] Chia H.N., Wu B.M. (2015). Recent advances in 3D printing of biomaterials. J. Biol. Eng..

[bib5] Drury J.L., Dennis R.G., Mooney D.J. (2004). The tensile properties of alginate hydrogels. Biomaterials.

[bib6] Eghrari A.O., Riazuddin S.A., Gottsch J.D. (2015). Overview of the cornea: structure, function, and development. Prog. Mol. Biol. Transl. Sci..

[bib7] Farrell R., McCally R., Albert D., Jakobiec F. (2000). Corneal transparency. Principles and Practice of Ophthalmology.

[bib8] Golchet G., Carr J., Harris M.G. (2000). Why don't we have enough cornea donors? A literature review and survey. Optometry.

[bib9] Gouveia R.M., Connon C.J. (2013). The effects of retinoic acid on human corneal stromal keratocytes cultured in vitro under serum- free conditions. Invest. Ophthalmol. Vis. Sci..

[bib10] Gouveia R.M., Koudouna E., Jester J., Figueiredo F., Connon C.J. (2017). Template curvature influences cell alignment to create improved human corneal tissue equivalents. Adv. Biosys..

[bib11] Griffiths G.W., Płociniczak Ł., Schiesser W.E. (2016). Analysis of cornea curvature using radial basis functions - Part I: Methodology. Comput. Biol. Med..

[bib12] Guo Q., Phillip J.M., Majumdar S., Wu P.H., Chen J., Calderón-Colón X., Schein O., Smith B.J., Trexler M.M., Wirtz D., Elisseeff J.H. (2013). Modulation of keratocyte phenotype by collagen fibril nanoarchitecture in membranes for corneal repair. Biomaterials.

[bib13] Hinton T.J., Jallerat Q., Palchesko R.N., Park J.H., Grodzicki M.S., Shue H.J., Ramadan M.H., Hudson A.R., Feinberg A.W. (2015). Three-dimensional printing of complex biological structures by freeform reversible embedding of suspended hydrogels. Sci. Adv..

[bib14] Hogan M.J., Alvarado J.A., Weddell J.E. (1971). The Cornea. Histology of the Human Eye.

[bib15] Jakab K., Marga F., Norotte C., Murphy K., Vunjak-Novakovic G., Forgacs G. (2010). Tissue engineering by self-assembly and bio-printing of living cells. Biofabrication.

[bib16] Karamichos D., Hutcheon A.E.K., Zieske J.D. (2014). Reversal of fibrosis by TGF-β3 in a 3D in vitro model. Exp. Eye Res..

[bib17] Klöck G., Pfeffermann A., Ryser C., Gröhn P., Kuttler B., Hahn H.J., Zimmermann U. (1997). Biocompatibility of mannuronic acid-rich alginates. Biomaterials.

[bib18] Lee K.Y., Mooney D.J. (2012). Alginate: properties and biomedical applications. Prog. Polym. Sci..

[bib19] Li F., Carlsson D., Lohmann C., Suuronen E., Vascotto S., Kobuch K., Sheardown H., Munger R., Nakamura M., Griffith M. (2003). Cellular and nerve regeneration within a biosynthetic extracellular matrix for corneal transplantation. Proc. Natl. Acad. Sci. U. S. A..

[bib20] Mandrycky C., Wang Z., Kim K., Kim D.H. (2016). 3D bioprinting for engineering complex tissues. Biotechnol. Adv..

[bib21] Meek K.M., Knupp C. (2015). Corneal structure and transparency. Prog. Retin. Eye Res..

[bib22] Mironov V., Reis N., Derby B. (2006). 3D bioprinting: a Beginning. Tissue Eng..

[bib23] Mironov V., Kasyanov V., Markwald R.R. (2011). Organ printing: from bioprinter to organ biofabrication line. Curr. Opin. Biotechnol..

[bib24] Muller L., Pels E., Vrensen G. (2001). The specific architecture of the anterior stroma accounts for maintenance of corneal curvature. Br. J. Ophthalmol..

[bib25] Murphy S.V., Atala A. (2014). 3D bioprinting of tissues and organs. Nat. Biotechnol..

[bib26] Ozbolat I.T., Yu Y. (2013). Bioprinting toward organ fabrication: challenges and future trends. IEEE Trans. Biomed. Eng..

[bib27] Rafat M., Li F., Fagerholm P., Lagali N.S., Watsky M.A., Munger R., Matsuura T., Griffith M. (2008). PEG-stabilized carbodiimide crosslinked collagen–chitosan hydrogels for corneal tissue engineering. Biomaterials.

[bib28] Ruberti J.W., Zieske J.D. (2008). Prelude to corneal tissue engineering - gaining control of collagen organization. Prog. Retin. Eye Res..

[bib29] Schneider C.A., Rasband W.S., Eliceiri K.W. (2012). NIH Image to ImageJ: 25 years of image analysis. Nat. Methods.

[bib30] Simonini I., Pandolfi A. (2015). Customized finite element modelling of the human cornea. PLoS One.

[bib31] Whitcher J.P., Srinivasan M., Upadhyay M.P. (2001). Corneal blindness: a global perspective. Bull. World Health Organ..

[bib32] Wright B., Cave R.A., Cook J.P., Khutoryanskiy V.V., Mi S., Chen B., Leyland M., Connon C.J. (2012). Enhanced viability of corneal epithelial cells for efficient transport/storage using a structurally modified calcium alginate hydrogel. Regen. Med..

[bib33] Zhang X., Zhang Y. (2015). Tissue engineering applications of three-dimensional bioprinting. Cell Biochem. Biophys..

